# A Novel Orthopaedic Resident Education Curriculum: Implementation of the Academy of Orthopaedic Surgeons Resident Orthopaedic Core Knowledge Platform

**DOI:** 10.2106/JBJS.OA.26.00047

**Published:** 2026-09-09

**Authors:** Joshua T. Bram, Mark F. Megerian, Austin C. Kaidi, Daniel W. Green, Duretti T. Fufa

**Affiliations:** 1 Hospital for Special Surgery, New York, New York

## Abstract

**Background:**

Traditional orthopaedic residency educational approaches rely on unstandardized lecture series and self-directed study, which can lead to inconsistencies in knowledge acquisition. The Residency Orthopaedic Core Knowledge (ROCK) curriculum, introduced by the American Academy of Orthopaedic Surgeons in 2022, is a structured, comprehensive framework designed to standardize orthopaedic education. This study evaluates resident satisfaction with implementation of the ROCK curriculum within a single orthopaedic residency program and its association with resident performance on the Orthopaedic In-Training Examination (OITE).

**Methods:**

A 2-year residency didactics curriculum was designed to incorporate the ROCK curriculum at a single, urban academic orthopaedic residency program with a wider breadth of topics starting in the 2023 to 2024 academic year. To assess pre-/post-implementation response to this change, a survey assessing resident attitudes toward lectures/study patterns was designed. This was administered at the end of the 2022-23 academic year (pre-ROCK), with responses also collected 1 (2023-24) and 2 (2024-25) years after implementation. The survey captured resident demographics, attendance patterns, content satisfaction, and study habits. Questionnaire answers and OITE scores were compared between the year 2022 to 2023 (pre-ROCK) and the years 2023 to 2025 (post-ROCK).

**Results:**

A total of 78 responses were recorded over the 3 years (63% male) out of a potential 135 respondents. The proportion of residents who agreed that conferences were high quality (93% vs. 70%, p = 0.013) and were satisfied with conferences (91% vs. 65%, p = 0.006) was greater with the ROCK curriculum. In addition, residents felt there was a more appropriate balance of case-/didactic-based lectures after implementation (86% vs. 57%, p = 0.006). With the new curriculum, program OITE percentile increased from 68th percentile in 2022 (pre-ROCK) to 79th percentile in both 2023 and 2024 to 83rd percentile in 2025.

**Conclusions:**

Integrating the ROCK curriculum into an orthopaedic residency didactics curriculum led to high resident satisfaction and an overall trend toward higher OITE scores. These results support the use of the ROCK curriculum to augment resident education. Orthopaedic program directors and education leaders alike can replicate or adapt our proposed lecture schedule.

## Introduction

Orthopaedic surgery residents are expected to master an expanding body of knowledge while simultaneously developing operative skills and clinical judgment. The Accreditation Council for Graduate Medical Education (ACGME) and American Board of Medical Specialties’ outline 6 Core Competencies, including excellence in patient care, resident knowledge, interpersonal and communication skills, professionalism, practice-based learning and improvement, and systems-based practice^1^. Traditional educational approaches in residency rely on nonstandardized lecture series, self-directed study, and variable clinical exposure, which can lead to inconsistencies in knowledge acquisition across training programs. These historical norms lacked universal guidelines for curriculum standards. As competency-based medical education continues to evolve, there is increasing emphasis on standardized curricula that align educational content with measurable outcomes.

The Residency Orthopaedic Core Knowledge (ROCK) curriculum was developed by the American Academy of Orthopaedic Surgeons (AAOS) in 2022 as a comprehensive educational framework designed to provide standards for orthopaedic resident learning^2^. The curriculum organizes core topics across orthopaedic domains aligned with examination-relevant content and modern educational principles, including spaced repetition, modular learning, and regular assessment. Furthermore, content was created by subject experts in the field that is linked to supplementary resources for further study. The AAOS, who maintains the content, has also indicated that the 545+ educational chapters will be updated on continuous cycle to reflect current, relevant information. By providing consistent exposure to essential topics and facilitating directed study, it aims to reduce variability in resident preparation and improve knowledge retention.

The Orthopaedic In-Training Examination (OITE) is a widely used objective benchmark of resident knowledge and serves as an important tool for both resident self-assessment and program evaluation. Performance on the OITE is associated with later success on the American Board of Orthopaedic Surgery (ABOS) Part 1 Certification Examination, underscoring the connection between resident education, OITE performance, and board preparedness^3^. As such, program directors have a vested interest in maximizing formal resident education/performance to graduate residents who are equipped to pass board examinations and enter independent practice.

While the ROCK curriculum serves as an invaluable repository of information for orthopaedic residencies, objective assessment of its effect on resident education remains limited. This study evaluates both resident satisfaction with didactics and its association with performance on the OITE after implementation of the ROCK curriculum within an orthopaedic residency program. We hypothesized that adoption of a structured curriculum would correlate with measurable improvements in OITE scores, reflecting enhanced knowledge acquisition and educational consistency.

## Materials and Methods

**Fig. 1:**
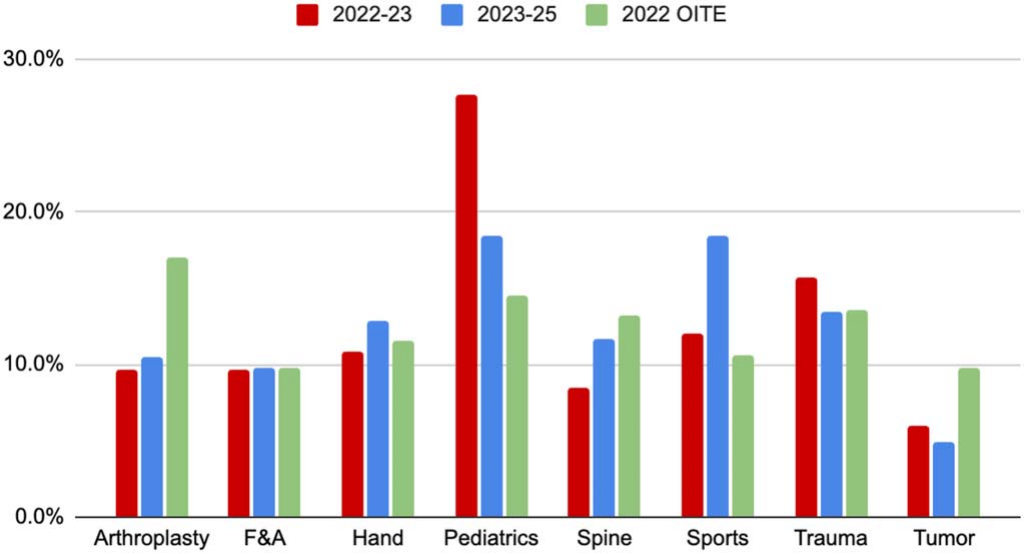
OITE topic distribution by subspecialty. OITE = Orthopaedic In-Training Examination.

Before the academic year 2023 to 2024, core resident didactics at our institution were composed of Monday and Tuesday, resident- and faculty-led lectures that followed a subspecialty-based, flipped classroom (i.e. residents reviewed preparatory materials ahead of time) model which repeated annually. Toward the end of the 2022 to 2023 academic year (Spring 2023), the authorship group reviewed the didactics curriculum for both structure and content. The prior 1-year curriculum was found to have poor alignment with the distribution of topics on the 2022 OITE, with few lectures on foundational orthopaedic principles (Appendix 1) and a disproportional focus on pediatrics (Fig. 1).

In the same academic year, the AAOS had released the ROCK curriculum. A new 2-year curriculum with a wider breadth of topics within each specialty was therefore designed to follow the ROCK chapters (Appendix 2). The main purpose of the change was to improve resident orthopaedic education and align our curriculum with this new program created by AAOS. Additional objectives of the didactics were to include topics with associated ROCK prereading, have faculty-led lectures whenever possible, and add in 4 weeks of specific OITE preparation per year. To assess the pre- and post-implementation responses to this curricular change at a single institution, an anonymous, voluntary survey assessing resident attitudes toward lectures and study patterns was designed based on similar prior studies (Table I)^4-8^. This was first administered at the end of the 2022-23 academic year, with responses compared with those obtained 1 and 2 years after implementation (end of 2023-24 and 2024-25 academic years, respectively). Each year, 45 residents were eligible to complete the survey, totaling 135 response opportunities. The curriculum and survey evaluating its implementation were developed independently of the AAOS and received no funding. Our program was also not involved in ROCK development or beta testing.

**Table I:** Resident ROCK Questionnaire

Question	Response options
Sex	Male
	Female
	Prefer not to say
Post-graduate year	1-5
While on institutional rotations, indicate the proportion for each attendance type (please list percent out of 100)	Attend in-person
	Attend virtually
	Miss due to clinical duties
	Skip
1. The conferences at our institution are generally of high quality. 2. Overall, I am satisfied with the educational conferences at my program. 3. I have interfaced with the ROCK curriculum enough to assess it. 4. The ROCK curriculum is easy to access. 5. The ROCK curriculum is well organized. 6. The ROCK curriculum is comprehensive. 7. The ROCK curriculum is useful for OITE preparation.	Strongly agree Agree Neutral Disagree Strongly disagree
I consider the current balance of case-based/interactive conferences to didactic lectures to be…	Too many case-based
	Too many didactic
	Appropriate
	No opinion
Percentage I use the following resources to study for the OITE (please list percent out of 100).	Practice questions (e.g. Orthobullets, ResStudy)
	Textbooks
	Review articles
	Prepared clinical flash cards (e.g. Anki)
	Review prior institutional conference recordings
	Review website reading (e.g. Orthobullets)
	Formal website review course (e.g. Orthobullets study plan)
Estimated number of practice questions completed in the past calendar year.	Number of questions

OITE = Orthopaedic In-Training Examination, and ROCK = Residency Orthopaedic Core Knowledge.

In the survey, resident sex and postgraduate year (PGY) were documented. The proportional attendance patterns for conferences were estimated by respondents as one of the following: attend in-person, attend virtually, miss due to clinical duties, or skip. Residents were then asked to rate on a 5-point Likert scale (from strongly disagree to strongly agree) several statements on conferences at our institution (Table I). They were also asked to estimate their number of study questions completed in the prior year. For the years 2022-23 and 2023-24, respondents were also asked to respond to the following prompts: “The ROCK curriculum is easy to access,” “The ROCK curriculum is well organized,” “The ROCK curriculum is comprehensive,” and “The ROCK curriculum is useful for OITE preparation.” Last, residents were asked to estimate the proportion of various OITE study materials, including Practice questions (e.g. Orthobullets [Lineage Medical, Inc.], ResStudy [AAOS]), Textbooks, Review articles, flashcards (e.g. Anki), prior conference recordings, website reading, and formal online review courses.

The overall framework of the study followed that of the Kirkpatrick model—a widely-used 4-level framework for evaluating the results of training and learning programs—which is commonly used in medical training assessment^9^. The model describes 4 levels: level 1 is reaction (e.g. satisfaction, feedback), level 2 is learning (e.g. knowledge, skills, attitude), level 3 is behavior (application, change), and level 4 is results (organizational outcomes). The pre-implementation questionnaire administered was meant to capture level 1 of the model, whereas the post-administration questionnaires reflected levels 2 and 3 (attitude towards the curriculum, behavioral changes in study habits). Program OITE scores then reflected both levels 2 and 4 (assessing both learning and the impact of the curriculum on overall scores). Questionnaire answers and OITE scores were compared between the academic year 2022 to 2023 (pre-ROCK) and the averages across 2023 to 2025 (post-ROCK). Categorical variables were compared with Fisher’s exact or chi-squared tests, as appropriate. Continuous variables were compared using independent t-tests. All statistical analyses were conducted using IBM SPSS Statistics for Macintosh (version 24.0) with significance set at p ≤ 0.05.

## Results

A total of 78 responses were recorded over the 3 years (63% male) capturing residents in all postgraduate years and 58% of all eligible residents during the study period (Table II). The numbers of responses by academic year (and proportion of eligible respondents) were 23 (51%), 22 (49%), and 33 (73%). Most respondents reported attending conference in-person across all years (73-83%), and the most common methods of studying for the OITE remained practice questions (56-68%), website reading such as Orthobullets (12-19%), and formal online courses (4-9%). The average number of practice questions completed was not significantly different between 2022-23 and 2023-25 (1,335 vs. 889).

**Table II:** Resident Education Responses

	2022-23	2023-24	2024-25
N	23	22	33
Sex			
Male	16 (70%)	13 (59%)	20 (61%)
Female	7 (30%)	9 (41%)	13 (39%)
PGY			
1	0 (0%)	0 (0%)	6 (18%)
2	5 (22%)	7 (32%)	4 (12%)
3	8 (35%)	6 (27%)	8 (24%)
4	6 (26%)	5 (23%)	10 (30%)
5	4 (17%)	4 (18%)	5 (15%)
Attendance type			
In-person	82%	83%	73%
Virtual	14%	10%	17%
Miss due to clinical	4%	7%	8%
Skip	0%	0%	1%
OITE study resources			
Practice questions	68%	64%	56%
Textbooks	3%	4%	12%
Review articles	3%	1%	2%
Flash cards	3%	8%	3%
Conference videos	1%	1%	3%
Website reading	12%	17%	19%
Formal course	9%	5%	4%
Average Questions	889 ± 880	925 ± 896	1,608 ± 1,634

OITE = Orthopaedic In-Training Examination, and PGY = postgraduate year.

When compared with the pre-ROCK curriculum, the proportion of residents who agreed or strongly agreed that conferences were high quality increased from 70% to 93% (p = 0.013). Resident satisfaction with educational conferences also increased from 65% to 91% (p = 0.006). As expected, the proportion of residents who reported they had interfaced enough with the ROCK curriculum to assess it increased from 35% to 71% (p = 0.003, Table III). In addition, residents felt there was a more appropriate balance of case-/didactic-based lectures with the new curriculum (86% vs. 57%, p = 0.006). Across the years 2022-23 and 2023-24, 88%, 88%, 94%, and 79% agreed or strongly agreed that the ROCK curriculum was easy to access, well-organized, comprehensive, and useful for OITE preparation, respectively.

**Table III:** ROCK Conference Satisfaction

	2022-23	2023-24	2024-25
N	23	22	33
Conferences high quality			
Strongly agree	1 (4%)	10 (45%)	13 (39%)
Agree	15 (65%)	12 (55%)	16 (48%)
Neutral	5 (22%)	0 (0%)	3 (9%)
Disagree	2 (9%)	0 (0%)	0 (0%)
Strongly disagree	0 (0%)	0 (0%)	1 (3%)
Satisfied with conferences			
Strongly agree	1 (4%)	10 (45%)	15 (45%)
Agree	14 (61%)	12 (55%)	13 (39%)
Neutral	5 (22%)	0 (0%)	3 (9%)
Disagree	3 (13%)	0 (0%)	1 (3%)
Strongly disagree	0 (0%)	0 (0%)	1 (3%)
Interfaced with ROCK			
Strongly agree	3 (13%)	7 (32%)	7 (21%)
Agree	5 (22%)	10 (45%)	15 (45%)
Neutral	6 (26%)	2 (9%)	6 (18%)
Disagree	6 (26%)	3 (14%)	4 (12%)
Strongly disagree	3 (13%)	0 (0%)	1 (3%)
Balance of case-based			
Too many case-based	1 (4%)	0 (0%)	1 (3%)
Too many didactic	8 (35%)	2 (9%)	2 (6%)
Appropriate	13 (57%)	20 (91%)	27 (82%)
No opinion	1 (4%)	0 (0%)	3 (9%)
Curriculum easy to access			
Strongly agree	7 (50%)	10 (53%)	—
Agree	5 (36%)	7 (37%)	—
Neutral	1 (7%)	1 (5%)	—
Disagree	1 (7%)	1 (5%)	—
Strongly disagree	0 (0%)	0 (0%)	—
Curriculum well-organized			
Strongly agree	9 (64%)	8 (42%)	—
Agree	3 (21%)	9 (47%)	—
Neutral	2 (14%)	2 (11%)	—
Disagree	0 (0%)	0 (0%)	—
Strongly disagree	0 (0%)	0 (0%)	—
Curriculum comprehensive			
Strongly agree	8 (57%)	11 (58%)	—
Agree	5 (36%)	7 (37%)	—
Neutral	1 (7%)	0 (0%)	—
Disagree	0 (0%)	1 (5%)	—
Strongly disagree	0 (0%)	0 (0%)	—
Useful for OITE preparation			
Strongly agree	3 (21%)	5 (26%)	—
Agree	9 (64%)	9 (47%)	—
Neutral	2 (14%)	5 (26%)	—
Disagree	0 (0%)	0 (0%)	—
Strongly disagree	0 (0%)	0 (0%)	—

OITE = Orthopaedic In-Training Examination, and ROCK = Residency Orthopaedic Core Knowledge

Following implementation of the ROCK curriculum, overall program OITE percentile scores showed a consistent positive trend, increasing from the 68th percentile in 2022 to the 79th percentile in both 2023 and 2024 to the 83rd percentile in 2025. In addition, the program demonstrated small but consistent improvements in the delta (i.e. difference) between program average OITE scores and percentiles vs. national ACGME averages across all PGY levels (Table IV), suggesting a weakly positive curriculum effect.

**Table IV:** Average Program OITE Scores and Percentiles Relative to National ACGME Means

	2022		2023-25	
PGY	Δ Score	Δ %	Δ Score	Δ %
1	3.0	1.0	5.0	2.0
2	3.0	2.0	5.3	2.0
3	3.0	1.0	3.7	1.7
4	5.0	2.0	8.3	3.0
5	-2.0	0.0	3.7	1.0
Average*	2.4	1.2	5.2	1.9

Δ = difference between average program OITE score/percentile and average national score/percentile.

OITE = Orthopaedic In-Training Examination, and PGY = postgraduate year.

* Formal statistical analysis of year-over-year program OITE scores could not be conducted based on these data, though there was an overall positive trend in scores (unclear statistical significance).

## Discussion

In this study, we found that implementation of a residency didactics program based on the ROCK curriculum improved satisfaction with core didactics. Residents reported didactics were of high quality and had an appropriate balance of case-based and didactic-based lectures. There was also an increase in average OITE score performance and percentiles vs. ACGME national averages. These results overall suggest a positive influence of the ROCK curriculum in supporting resident education and OITE performance.

Interest in orthopaedic surgical education has been evolving^10^. Martinez et al. reviewed 383 articles pertaining to orthopaedic surgical education over a 6-year period (2016-2021) and found that 70% related specifically to orthopaedic resident education^11^. The most common topic was resident curriculum, highlighting interest in evaluating resident didactics experiences. While clinical skills are paramount, a strong fund of knowledge during residency is predictive of future board success. There is a clearly identifiable link between OITE performance, a proxy for resident knowledge, and performance on board certification examinations. Since 2020, the AAOS and ABOS have connected the OITE and ABOS Part 1 Certification Examination to identify minimum OITE scores that would translate to a passing score on Part 1, known as a “linking score.” In assessing the OITE linking score, Fritz et al. demonstrated positive predictive values of 99.8% and 99.7% in 2022 and 2023, indicating that for those with an OITE score at/above the linking score, more than 99% then passed the Part 1 Certification Examination in those years^3^. Several other studies have demonstrated a similar association between lower OITE scores and risk for failing written board examinations^3,12,13^, underscoring the importance of maximizing in-training didactics to adequately prepare residents for their written boards.

The ROCK curriculum represents an intentional measure to achieve that end. Our study not only provides credence to the use of the ROCK curriculum for resident education but also provides a framework for other programs. As illustrated in Appendix 2, lecture topics are effectively spread out over a 2-year timeline and organized by specialty. Within each lecture topic, the relevant ROCK chapters are embedded for faculty and residents to review before lectures, while also serving as a reference for later use. After implementation of the ROCK curriculum at our institution, residents more strongly agreed that conferences were of high quality (93% vs. 70%) and were more satisfied with education content (91% vs. 65%) than prior years. In addition, >90% of residents found the ROCK curriculum to be comprehensive in nature.

Though not captured in our survey, residents anecdotally found the embedded ROCK chapter links very convenient to later reference for review purposes. Residency directors and other orthopaedic education leaders can replicate or adapt our institution’s lecture schedule for use in their respective programs. We recommend, based on our experiences with this new curriculum, having faculty from each specialty present on the topic when possible to maximize resident engagement as it has been both shown that junior lecturers (i.e. given from fellow residents) and traditional lecture–based conference styles are negative predictors of perceived conference value by orthopaedic residents^14^. Our findings suggest that the ROCK curriculum implemented at our program improved resident engagement. Aside from the impact on residents, we have found that providing faculty lecturers with the ROCK topics and resources facilitates composition of high-yield, resident-level lectures as they are provided focused formal preparatory materials. Faculty lecturers can also use the predetermined topics to influence case-based examples. This is supported by our finding that residents found a more appropriate balance of case-based lectures after the implementation of the ROCK curriculum.

This study has several weaknesses. Most importantly, this was an observational survey study of a single residency program’s experience with the new ROCK curriculum. This relied on accurate resident reporting at the end of each academic year, which is subject to recall bias. While our study successfully surveyed the majority of our program’s residents, our results are limited to the responses provided and may be subject to selection bias because survey and study participation were voluntary: 23 residents (51%) in 2022-23, 22 residents (49%) in 2023-24, and 33 residents (73%) in 2024-25. In addition, our program is an academic program at an urban, tertiary care, specialty orthopaedic hospital, perhaps limiting the generalizability of our results to more community-based or rural programs. Therefore, assessing the influence of the ROCK curriculum across other program types (e.g. community-based, hybrid [community + academic affiliation] or osteopathic) would be beneficial. Future studies assessing implementation of this curriculum at such programs is therefore necessary. While introduction of the ROCK curriculum was associated with an increase in OITE performance, this is simply a correlation and other factors such as increased emphasis on resident OITE preparation and performance by program leadership may have also had an impact. In the new curriculum, 4 dedicated weeks of OITE preparation were incorporated annually, though overall institutional emphasis on OITE performance did not formally change. The fact that the self-reported the number of OITE practice questions performed and studying modes were similar between pre- and post-ROCK resident cohorts in our study do, however, support the importance of the ROCK curriculum introduction. In addition, a formal statistical analysis of year-over-year program OITE scores could not be conducted based on these single data points, though there was an overall positive trend in scores (unclear statistical significance). Beyond correlation to OITE performance, importantly, resident overall satisfaction with the didactics curriculum increased following implementation of the ROCK curriculum.

## Conclusion

Integrating the ROCK curriculum into an orthopaedic surgery resident education series improved already high resident satisfaction with educational conferences, resulting in an overall trend toward higher OITE score performance and program percentile rank. These results support the use of the ROCK curriculum to augment resident education. Orthopaedic surgery program directors and education leaders alike can replicate or adapt our proposed lecture schedule with the corresponding ROCK chapters/links in their respective programs. If the ROCK curriculum becomes more ubiquitous, future research should further isolate its effect on OITE and ABOS board examination performance as a proxy for resident knowledge.

## Conflicts of Interest

Joshua Bram, Mark Megerian, and Austin Kaidi have no disclosures to report. Daniel Green: Orthopediatrics: IP Royalties; Patella Femoral Foundation NY State Society of Orthopedic Surgeons: Type: Board of Directors or committee member Self. Duretti Fufa: Integra: Paid consultant; Integra Life Sciences: Research support; Medartis: Paid consultant.

## Appendix

Supporting material provided by the authors is posted with the online version of this article as a data supplement at jbjs.org (https://links.lww.com/JBJSOA/B326). This content was not copyedited or verified by JBJS.
